# A Snapshot of a Coral “Holobiont”: A Transcriptome Assembly of the Scleractinian Coral, *Porites*, Captures a Wide Variety of Genes from Both the Host and Symbiotic Zooxanthellae

**DOI:** 10.1371/journal.pone.0085182

**Published:** 2014-01-15

**Authors:** Chuya Shinzato, Mayuri Inoue, Makoto Kusakabe

**Affiliations:** 1 Marine Genomics Unit, Okinawa Institute of Science and Technology Graduate University, Onna, Okinawa, Japan; 2 Atmosphere and Ocean Research Institute, The University of Tokyo, Kashiwa, Chiba, Japan; Auburn University, United States of America

## Abstract

Massive scleractinian corals of the genus *Porites* are important reef builders in the Indo-Pacific, and they are more resistant to thermal stress than other stony corals, such as the genus *Acropora*. Because coral health and survival largely depend on the interaction between a coral host and its symbionts, it is important to understand the molecular interactions of an entire “coral holobiont”. We simultaneously sequenced transcriptomes of *Porites australiensis* and its symbionts using the Illumina Hiseq2000 platform. We obtained 14.3 Gbp of sequencing data and assembled it into 74,997 contigs (average: 1,263 bp, N50 size: 2,037 bp). We successfully distinguished contigs originating from the host (*Porites*) and the symbiont (*Symbiodinium*) by aligning nucleotide sequences with the decoded *Acropora digitifera* and *Symbiodinium minutum* genomes. In contrast to previous coral transcriptome studies, at least 35% of the sequences were found to have originated from the symbionts, indicating that it is possible to analyze both host and symbiont transcriptomes simultaneously. Conserved protein domain and KEGG analyses showed that the dataset contains broad gene repertoires of both *Porites* and *Symbiodinium*. Effective utilization of sequence reads revealed that the polymorphism rate in *P. australiensis* is 1.0% and identified the major symbiotic *Symbiodinium* as Type C15. Analyses of amino acid biosynthetic pathways suggested that this *Porites* holobiont is probably able to synthesize most of the common amino acids and that *Symbiodinium* is potentially able to provide essential amino acids to its host. We believe this to be the first molecular evidence of complementarity in amino acid metabolism between coral hosts and their symbionts. We successfully assembled genes originating from both the host coral and the symbiotic *Symbiodinium* to create a snapshot of the coral holobiont transcriptome. This dataset will facilitate a deeper understanding of molecular mechanisms of coral symbioses and stress responses.

## Introduction

Coral reefs are estimated to harbor roughly one-third of all described marine species, and their productivity supports approximately one-quarter of marine fisheries. The major architects of coral reefs, the scleractinian corals, are anthozoan cnidarians that form obligate endosymbioses with photosynthetic dinoflagellates of the genus *Symbiodinium*. The unicellular symbionts are harbored in the host coral's gastrodermal (endodermal) tissue in intracellular vacuoles known as symbiosomes [1], which are thought to originate from the plasma membrane of host cells during the initial acquisition of symbionts by a phagocytic process [2]. Although many of the details of the interaction between the host and *Symbiodinium* remain to be explored, this association enables the massive rates of calcification that distinguish reef-building corals from other anthozoans, such as sea anemones and zoanthids.

Coral reefs face a range of environmental changes, including ocean acidification, seawater temperature increases, and declines in coral abundance. Extensive loss of reef habitats is one of the most pressing environmental issues of our time [3]–[5]. Recently, increasing instances of “coral bleaching” have been observed. In most cases, coral bleaching is a breakdown of the mutualism between the coral and the photosynthetic dinoflagellate, resulting from a stress response to environmental perturbation. The integrity of the coral holobiont – a complex symbiosis between the coral animal, its endosymbiotic zooxanthellae, and an associated community of microorganisms is essential for maintaining coral health [6]. The molecular mechanisms underlying the collapse of symbiosis and the bleaching response are complex and are still little understood. Bleaching is currently viewed as a host response to a compromised symbiont that is analogous to the innate immune responses that occur in other host–microbe interactions. Expulsion or elimination of the symbiont from host tissues is thought to involve a variety of mechanisms, including exocytosis, host cell detachment, and host cell apoptosis [7].

Genomic information for cnidarians has been accumulating; the genomes of two non-symbiotic cnidarians, the anemone *Nematostella vectensis* (Anthozoa) [8], and a hydra, *Hydra magnipapillata* (Hydrozoa) [9], have been sequenced. The genome of a scleractinian coral, *Acropora digitifera*, was decoded using next-generation sequencing (NGS) technology [10]. The latter genome was estimated to comprise 420 Mbp and contains about 23,700 predicted protein-coding genes. Recently NGS-based transcriptome datasets have become available for several anthozoan cnidarians including several coral species: *Acropora millepora* [11], [12], *A. palmata* [13] and *Pocillopora damicornis* [14], and a sea anemone *Aiptasia pallida* [15]. In addition, transcriptomes of two cultured strains of *Symbiodinium* have been reported [16] and the nuclear genome of *S. minutum* was decoded [17]. In order to better understand the molecular interactions, it is worthwhile to simultaneously capture the molecular states of all organisms comprising a coral holobiont.

The massive stony corals of the genus *Porites* are common, important reef builders in the Indo-Pacific Ocean. More than 80 named species and numerous unclassified forms have been identified [18]. In contrast to *Acropora* species, *Porites* corals generally transmit their symbionts directly from parents to offspring (vertical transmission) rather than acquiring them anew from the environment (horizontal transmission) in each generation [19]. *Porites* species have thicker tissues and appear more robust to thermal stress than other corals, such as *Acropora*, that have thinner tissues [20]. Thus *Porites* corals have been used for comparative analyses of stress responses [21]. Laboratory studies indicate that acidified seawater reduces calcification of *Porites* corals [22]–[24]. Geochemical tracers, such as oxygen isotope ratios, strontium-calcium ratios, and heavy metal concentrations in growth rings of the CaCO_3_ *Porites* skeleton have been used to monitor changes in sea surface temperature, salinity, and/or marine pollutants [25]–[28]. By such means, *Porites* taxa have been used to reconstruct past environmental changes so as to better understand tropical climate systems and to predict climate change [29], [30]. Nevertheless, molecular information about *Porites* corals is still limited. To address this deficiency and to improve the utility of *Porites* as an environmental indicator, we sequenced the transcriptome of *Porites australiensis* and its symbiotic algae (Figure? 1) using next generation technology (Illumina HiSeq2000) and constructed a transcriptome dataset that contains a large proportion of a symbiotic alga.

**Figure 1:**
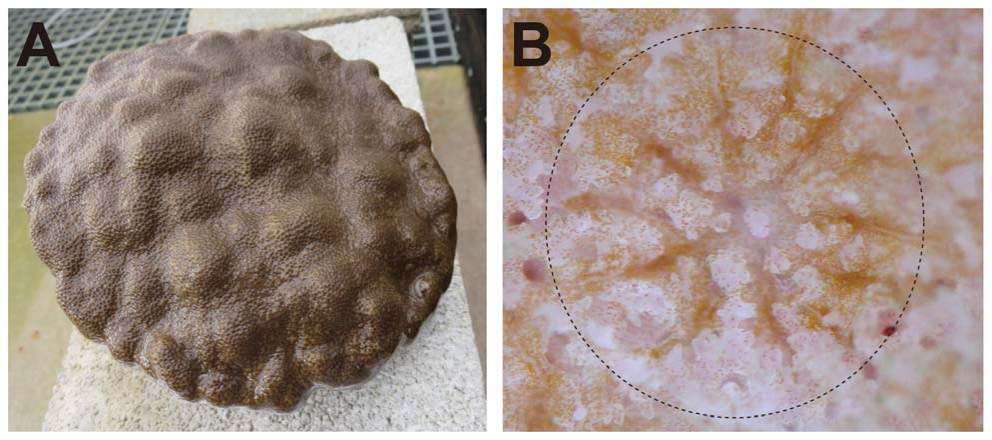
The scleractinian coral, *Porites australiensis*. (A) A *P. australiensis* colony used in this study. The diameter is approximately 20 cm. (B) High magnification photo of the *P. australiensis*. Dot circle indicates one polyp and tiny brown particles are symbiotic algae, dinoflagellates, *Symbiodinium*.

## Materials and Methods

### RNA isolation, Transcriptome sequencing and assembly

The coral sample used in this study was collected at Sesoko island, Okinawa, Japan, under the Okinawa prefecture permission (Number: 20–69). A small *Porites australiensis* colony that has been maintained in the Sesoko research station at the University of the Ryukyus for five years (Figure? 1) was used in this study. A fragment (2 cm diameter) from the coral was snap frozen in liquid nitrogen and pulverized with an iron mortar and pestle. Total RNA was isolated using an RNeasy RNA extraction kit (QIAGEN). Total RNA was then fragmented into about 200 bp lengths and an RNA-seq library was prepared using a TruSeq RNA Sample Prep Kit v2 (Illumina). cDNA normalization using a duplex-specific nuclease was also performed. The library was sequenced (100 bp paired-end reads) using the Illumina HiSeq 2000 platform. Library preparation, cDNA normalization, and sequencing were outsourced to Hokkaido System Science Corporation, Japan. Low quality bases (Phred quality value, QV≥20) were trimmed from the raw data and read pairs of at least 80 bp were retained using SolexaQA [31]. Possible PCR duplicates that originated during library preparation were removed with ConDeTri [32]. Contamination with TruSeq adapter sequences was removed by ea-utils (http://code.google.com/p/ea-utils). Subsequently high-quality paired end reads were assembled with Velvet/Oases software [33], [34] using different K-mer sizes [35], [45], [55] and merged by K-mer 27 using Oases [34]. For each word size, the longest isoform of each locus was selected. In addition, in order to recover low-expression genes and to produce longer assemblies, different coverage cut-off values (5, 8, 10 and 12) were also applied. Contigs originating from different coverage cutoff values were concatenated and redundant sequences and transcript-variants were removed by CDHIT-EST [35] using default parameters. Contigs over 200 bp length were retained. Additionally, contigs containing translated Open Reading Frames (ORFs) in which 95% of the amino acids were identical were considered duplicates. In such cases, one of the duplicates was removed using CDHIT [35].

### Separation of host or symbiont-originated sequences

To distinguish whether contig sequences originated from host coral or symbiont *Symbiodinium*, nucleotide sequences of the assembled contigs were aligned to the genome sequences of both coral *A. digitifera* [10] and dinoflagellate *S. minutum* [17] by BLASTN, and contigs that show nucleotide similarities with *A. digitifera* or *S. minutum* were identified. Contigs aligned with the both genomes were not annotated in this study. Contigs that aligned only to the *A. digitifera* genome were annotated as “*Porites* contigs” while those that aligned only to the *S. minutum* genome were annotated as “*Symbiodinium* contigs”, respectively. Different e-value cutoffs (1e^−1^, 1e^−2^, 1e^−3^, 1e^−4^, 1e^−5^ and 1e^−10^) were examined and a cutoff that maximized the number of *Porites* or *Symbiodinium* contigs was adopted (Table S3). We also applied the same analyses for reported coral transcriptome data from *A. millepora* [12], *A. palmata* [13], and *P. damicornis* [14], in order to investigate proportions of *Symbiodinium* sequences in each dataset.

### Annotation of transcriptome assembly

Assembled transcriptome data were annotated as follows: 1) by BLAST homology searches against public protein databases: NCBI non-redundant protein sequences (NR) and Swiss-Prot [36], 2) by assignment of Gene Ontology (GO) terms [37], 3) by mapping to pathways using the KEGG annotation service KAAS [38], and 4) conserved protein domain searches with Pfam [39]. BLASTX homology searches were conducted against the Swiss-Prot and NCBI NR protein databases and an e-value cutoff of 1e^−5^ was applied. For GO annotation, GO IDs were assigned based on the UniProt IDs of the best matches in the Swiss-Prot database [36]. Then Generic GO slim terms were assigned using map2slim.pl in go-perl (http://search.cpan.org/~cmungall/go-perl/). For KAAS pathway annotation and analysis, we used the bi-directional best-hit (BBH) method to query the set of organisms representative for ‘eukaryotes’ as suggested on the KAAS website, using default settings. To screen and identify conserved protein domains, we used the Pfam database (Pfam-A.hmm, release 24.0; http://pfam.sanger.ac.uk) [39]. Translated amino acid entries matching conserved domains were identified using HMMER searches (hmmer3) [40]. In order to avoid eliminating *Symbiodinium*- or coral-specific rapidly evolving domains, we used an e-value cutoff of 1e^−3^, as proposed by Kawashima et al. [41]. For assessing completeness of the *Porites* contigs, mutual best-hit blast analyses (TBLAST, BLASTX, 1e^−5^) were performed against the sea anemone *Nematostella vectensis* proteome [8] in order to identify possible orthologs between *Porites* and *Nematostella*. We used *Nematostella* as a reference proteome in order to compare number of orthologous pairs between *A. digitifera* [10] and *Nematostella* with those of *Porites* and *Nematostella*. Alignment coverage of each *Porites* contig across full-length amino acid sequence of its possible *Nematostella* ortholog were investigated based on the BLAST search. For *Symbiodinium* contigs, *S. minutum* proteome dataset [17] was used for identifying orthologous pairs.

### Estimation of polymorphism in Porites

For estimating the polymorphism rate of *P. australiensis*, high-quality, trimmed Illumina reads (see above) were re-mapped against possible host-originated contigs (*Porites* contigs) using the Burrows-Wheeler Aligner (BWA) [42] and SNPs (single nucleotide polymorphisms) in each contigs were detected. Because BWA allows gaps in alignments, we set the maximum indel size to 5 bp (bwa aln -n 0.05; since the maximum read length is 100 bp) in order to detect small indels. SNPs and small indels were called using SAMtools software packages [43]. To ensure reliable and high quality variant calling, SNPs and small indels were called only for positions with a minimal mapping quality (-Q) of 25, a coverage value (-d) of 10, and a maximum read depth (-D) of 200 using the varFilter command in the SAMtools package.

### Identification of the symbiotic Symbiodinium type

Illumina sequence data that originated from the *Symbiodinium* internal transcribed spacer 2 (ITS-2) region of the nuclear ribosomal array were identified using DNA sequences of *Symbiodinium*-specific ITS-2 primers used for genotyping: itsD and ITS-2rev2 [44]. Sequences reads containing these primer sequences were detected using BLAST (BLASTN) and 100% matched sequences were retrieved. Then these were subsequently assembled into a contig using Phrap [45] with default parameters. Sequence variations within the assembled ITS-2 sequence were checked with BWA [42] and SAMtools [43] as mentioned above.

## Results and Discussion

### Sequencing and assembly of the transcriptome

Total RNA was isolated from a single colony of *P. australiensis* (Figure? 1A), and then a normalized cDNA library was prepared and sequenced with the Illumina HiSeq2000 system. We obtained 71 million paired-end sequences (14.3 Gbp, Table S1). Raw sequence data were submitted to the DDBJ Sequence Read Archive (DRA) under accession number DRA000906 (BioProject ID: PRJDB731). Subsequently, 45 million quality-trimmed read pairs (QV≥20, both paired read ≥80 bp and PCR duplicates and TruSeq adapter sequences removed, approximately 9 Gbp, Table S1) were assembled using Velvet/Oases software (Figure? 2, Table? 1). Contigs of <200 bp were discarded because the RNA was fragmented into approximately 200-bp lengths during library preparation (see Material and Methods) and these short contigs were most likely to be truncated. After exclusion of redundant sequences and transcript variants, we obtained 74,997 contigs without ambiguous sequence gaps, totaling 94.73 Mbp (Figure? 2, Table? 2). Although the size distribution was weighted toward smaller contigs, there were 31,199 contigs with lengths >1,000 bp, 1,952 contigs >5,000 bp, and 198 contigs >10,000 bp, respectively (Figure? 3A). The maximum contig length reached 54,796 bp; the average was 1,263 bp, and the N50 size was 2,037 bp (Table? 2), indicating that the assembly is of comparable or better quality than extant anthozoan transcriptome assemblies using NGS platforms (Table S2). Assembled sequences have been submitted to the DDBJ/EMBL-Bank/GenBank Transcriptome Shotgun Assembly (TSA) Database under accession numbers FX435232-FX505330 and FX799345-FX804242.

**Figure 2:**
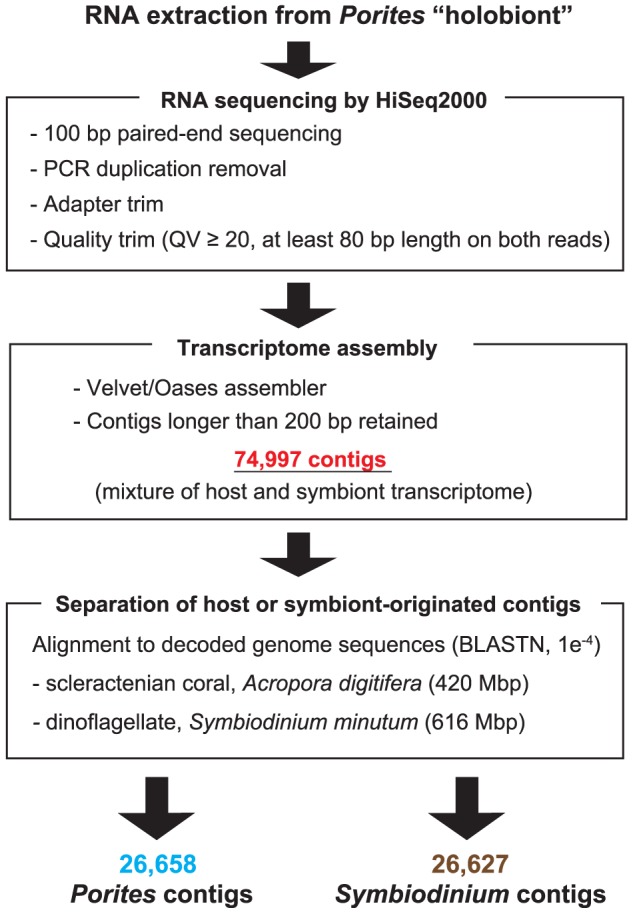
Flowchart of transcriptome sequencing, assembly, and separation of host or symbiont sequences.

**Figure 3:**
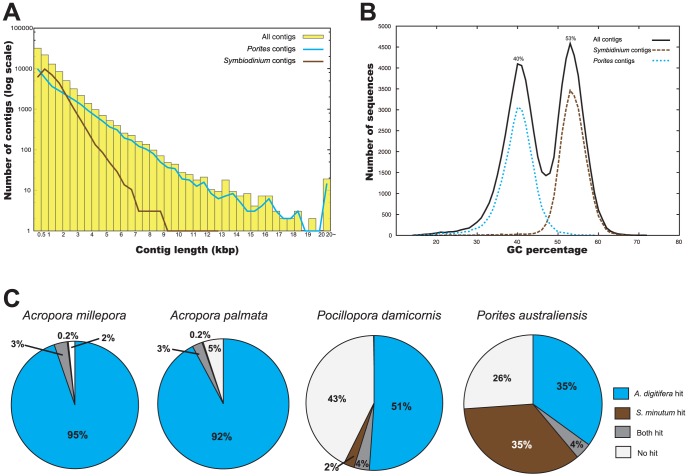
Analyses of the *Porites australiensis* “holobiont” transcriptome assembly. (A) Length distribution of transcripts in the transcriptome assembly. Yellow boxes indicate all contigs. Blue and brown lines indicate *Porites* contigs and *Symbiodinium* contigs, respectively. (B) Distribution of GC percentages of the assembled contigs. Black line: all contigs, blue dot line: *Porites* contigs, brown dot line: *Symbiodinium* contigs. (C) Comparison of proportions of *Symbiodinium* sequences between the scleractinian coral transcriptome assemblies. Each sequence was aligned to *A. digitifera* and *S. minutum* genome sequences by BLASTN (1e^−5^). Note that the high percentage of *A. digitifera* sequences shared with the *A. millepora* and *A. palmata* datasets occur because these three corals are congeneric.

**Table 1:** Summary of the *Porites australiensis* “holobiont” transcriptome assembly.

Number of unique sequences	74,997
Total basepair (Mbp)	94.73
Average (bp)	1,263
N50 size (bp)	2,037
Maximum contig length (bp)	54,796
Number of *Porites* cotigs	26,658 (35.5%)
Total basepair (Mbp)	44.57
Average (bp)	1,672
N50 size (bp)	2,960
Maximum contig length (bp)	44,581
Number of *Symbiodinium* contigs	26,627 (35.5%)
Total basepair (Mbp)	32.01
Average (bp)	1,202
N50 size (bp)	1,571
Maximum contig length (bp)	12,901

**Table 2:** Summary of annotations of the transcriptome assembly.

	Number of sequence	Number of hit genes by BLAST search		Pfam		KEGG	
		NCBI NR (BLASTX, 1e−5)	Swiss-Prot (BLASTX, 1e−5)	Number of domain	Number of annotated genes	Number of KO	Number of annotated genes
All	74,997	33,935 (45.2%)	25,893 (34.5%)	6,081	30,002 (40%)	5,001	7,452 (9.9%)
*Porites* contigs	26,658	14,206 (53.3%)	11,582 (43.4%)	4,528	11,857 (44.5%)	3,633	4,264 (16%)
*Symbiodinium* contigs	26,627	13,813 (62.4%)	10,240 (38.5%)	3,569	13,513 (50.7%)	1,352	1,854 (15.3%)

### Dissecting the “holobiont” transcriptome

Because total RNA was isolated from an adult *P. australiensis* colony (Figure? 1A), the transcriptome assembly contains genes from both corals and their zooxanthellae symbionts (Figure? 1B). When the GC content distribution of all contigs in the assembly was analyzed, two clear peaks of approximately 40% and 53% were detected (Figure? 3B), suggesting that these peaks possibly originated from the host (*P. australiensis*) and the symbiont (*Symbiodinium* sp.), respectively. The GC % of *A. digitifera* exons is about 40% [10] and that of a sea anemone, *Nematostella*, is similar [8], suggesting that a GC content of about 40% may be typical for anthozoan cnidarians. Therefore the 40% peak probably reflects *P. australiensis* genes. Taking into consideration the mean GC % of exons in the *Symbiodinium minutum* genome is about 50% [17], the secound peak might corresponds to the contigs from *Symbiodinium*.

Next we tried to identify contigs in the assembly that originated with *Porites* or *Symbiodinium*. We found that nucleotide alignment to both the *Acropora* and *Symbiodinium* genomes by BLASTN effectively separated them. In this study, we adopted an e-value cutoff 1e^−4^ (Table S3). 30,446 sequences hit against the *A. digitifera* genome and 30,415 sequences against *S. minutum* genome (3,788 sequences sequences overlapped) respectively (Table S3). We subsequently removed overlapping sequences, and annotated 26,658 sequences as “*Porites* contigs” and 26,627 sequences as “*Symbiodinium* contigs”. Annotated contigs comprised about 70% of all contigs and GC distributions of *Porites* and *Symbiodinium* contigs clearly matched the two peaks detected in the whole assembly (Figure? 3B), indicating that we effectively separated *Porites* and *Symbiodinium* genes in the assembly. These annotated contigs were used for further analyses (Table? 2, Figure? 3, 4).

**Figure 4:**
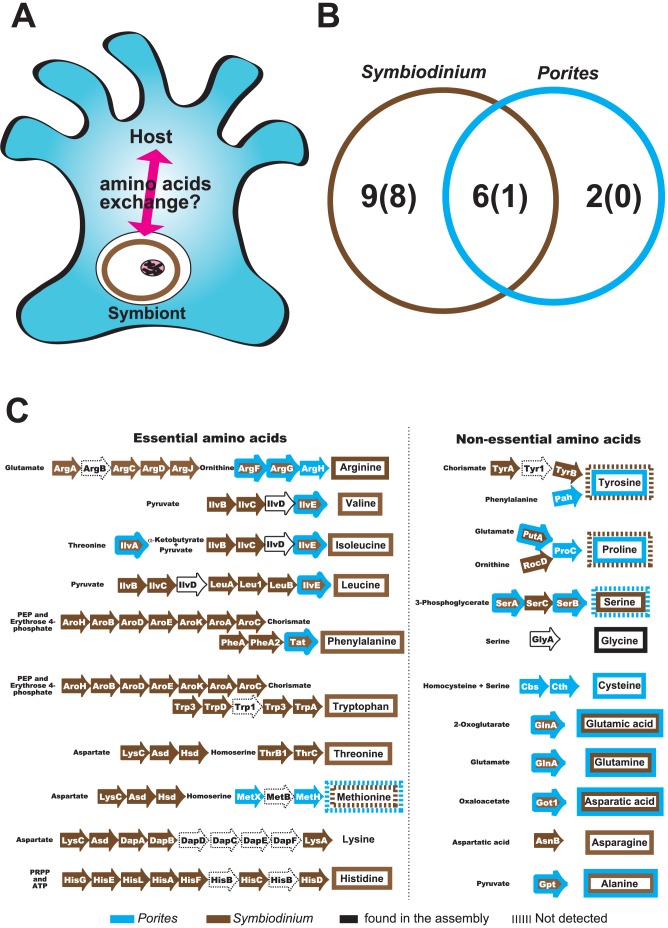
Amino acid metabolism in *Porites* holobiont. (A) Schematic drawing of amino acid exchange between a coral polyp and symbiotic *Symbiodinium*. (B) Summary of amino acid biosynthesic capabilities of *Porites* (host) and *Symbiodinium* (symbiont) inferred from KEGG annotation. Numbers indicate how many amino acids can be produced by *Porites* or *Symbiodinium*, and numbers in blankets show numbers of essential amino acids. Blue circle indicates *Porites* and brown circle indicates *Symbiodinium*, respectively. (C) Detail annotations of amino acid biosynthetic pathways in the *Porites* holobiont. Arrows indicate enzymes involved in each amino acid biosynthetic pathway. Enzymes that are not annotated as *Porites* or *Symbiodinium* are shown with a black line, and those not detected in the transcriptome dataset are shown with dotted lines. Blue indicates enzymes identified in *Porites* contigs. Brown indicates enzymes detected in *Symbiodinium* contigs. Brown arrows boxed in blue lines indicate enzymes detected in both *Porites* and *Symbiodinium*. Amino acids that can be produced by *Symbiodinium* are boxed in brown and those that can be produced by *Porites* are boxed in blue, respectively. Amino acids boxed in dotted lines indicate that biosynthetic capabilities are not clear.

In most transcriptome analyses of anthozoans [12], [13], [15], aposymbiotic materials were used for sequencing in order to minimize *Symbiodinium* contamination. Thus, we suspect that *Symbiodinium* sequences in those transcriptome assemblies have been under-detected. It has also been reported that RNA of adult *P. damicornis* colonies containing *Symbiodinium* was extracted, sequenced on a 454 platform and assembled, but that few *Symbiodinium* sequences had been isolated [14]. In contrast with previous studies, a large proportion of *Symbiodinium* sequences (at least 26,627 contigs, 35% of total assembled sequences) were in the transcriptome assembly (Figure? 3C). We confirmed that coral transcriptome data reported to date contained very few *Symbiodinium*-originated sequences (Figure? 3C). Therefore this study represents the first simultaneous transcriptome profile of both counterparts of coral holobiont.

### Transcriptome annotation

For transcriptome annotation, BLAST homology searches against protein databases (NCBI NR and Swiss-Prot) were performed. Among 74,997 contigs, 33,935 (45.2%) have significant similarities (BLASTX, 1e^−5^) with NCBI NR and 25,893 (34.5%) with Swiss-Prot database entries, respectively. As mentioned above, 30,446 sequences showed similarity to the *A. digitifera* genome and 30,415 sequences to the *S. minutum* genome, respectively (Table? 2). Taken together, we were able to annotate 60,516 (80.7%) sequences using public protein databases (NCBI NR and Swiss-Prot) and *A. digitifera* [10] and *S. minutum* [17] genomic data. The remaining 14,481 sequences (total 7.12 Mbp) could not be annotated by BLAST and their average length (491.7 bp) was much shorter than that of the total assembly. These may be fragmented regions of other contigs, assembly artifacts, or bacterial contaminants. Some show similarities to coral microsatellite sequences deposited in the NCBI nucleotide (NT) database (data not shown).

Conserved protein domains in the contigs were identified with hmmer3 [40] using the Pfam domain database [39]. We identified 6,081 Pfam domains in 30,002 contigs (Table? 2). We were able to assign 5,001 KEGG ORTHOLOGY (KO) IDs to 7,452 contigs (Table? 2) using the KAAS BBH method [38].

Based on a BLAST search against the Swiss-Prot database, we were able to assign at least one GO term to the 11,048 *Porites* and 9,915 *Symbiodinium* contigs, respectively. These 20,963 contigs were grouped into 101 categories in the second hierarchy of the Generic GO slim. Figures S1, S2, S3 show GO annotation results for three GO sub-ontologies (cellular component, biological process, and molecular function). Since *Porites* corals are multicellular animals, cell death, neurological system process, cell proliferation, cell adhesion, and cell-cell signaling terms are more heavily represented than in *Symbiodinium* (2× more, Figure S1). DNA binding, enzyme binding, and transcription factor binding activity also seem to be higher in *Porites* (Figure S2). These may reflect the fact that 464 *Porites* contigs possess transcription factor domains (Table S4), whereas only 28 *Symbiodinium* contigs do (data not shown). On the other hand, transcripts of genes related to photosynthesis, transport, and generation of energy are more abundant among *Symbiodinium* contigs (Figure S1).

### Estimating transcriptome completeness

About 44% of *Porites* contigs contain conserved protein domains and 4,528 unique Pfam domains were identified (Table? 2). In the gene models of the *A. digitifera* genome, unique 3,684 Pfam domains were detected [10], indicating that the *Porites* contigs probably contain a comparable variety of protein domains. In addition, we performed KEGG annotation and 3,633 unique KO IDs were identified among the *Porites* contigs (Table? 2). We detected 3,684 unique KO IDs in the *A. digitifera* proteome using the KAAS BBH method. A comparable number of KO IDs might indicate that, although RNA-seq was performed using a single RNA library, the majority of *P. australiensis* genes were successfully recovered in the transcriptome assembly. However it could be possible that those numbers in *Porites* contigs are overestimated due to incomplete clustering of transcript variants in the assembly.

An increasing number of studies have recently identified genes that encode transcription factors and signal transduction molecules in corals and have determined their roles in cnidarian evolution [46]–[50]. Therefore, for further assessing the completeness of the *Porites* transcriptome, the repertoire of transcription factors and signal transduction molecules was compared with those of other cnidarians whose genomes have been decoded (Table S4, 5). We detected comparable numbers of transcription factors and signaling molecules among the *Porites* contigs to what we previously found in the *A. digitifera* genome [10], e.g. *Porites*: 89 homeobox, 24 HMG box and 15 wnt genes; *A. digitifera*: 97 homeobox, 26 HMG box and 15 wnt genes (Table S4, 5). Next we identified orthologous gene pairs between *Porites* contigs and *Nematostella* by mutual-best-hit blast analysis; 7,825 pairs were detected. The similarities of the *A. digitifera* and *Nematostella* genomes resulted in 8,416 orthologous pairs, suggesting that *Porites* contigs represent the majority of the gene repertoire for *Porites australiensis*. 6,324 orthologous genes out of 7,825 have more than 80% alignment coverage across *Nematostella* orthologs, suggesting that most of the *Porites* contigs recover full-length open reading frames.

On the other hand, 3,569 Pfam domains and 1,352 KO IDs were detected among *Symbiodinium* contigs (Table? 2), while 4,035 Pfam domains and 2,622 KO IDs were detected in the *S. minutum* gene model [17], respectively. We also identified 28 *Symbiodinium* contigs containing transcription factor domains (data not shown). It has been reported that in transcriptome assemblies from two cultured strains of *Symbiodinium*, 156 and 87 sequences, respectively, contained at least one known transcription factor domain [16]. The *S. minutum* genome was found to contain 32 transcription factor genes [17]. We detected 15,914 orthologous pairs between the *Symbiodinium* contigs and *S. minutum* genome. In *S. minutum* genome, 23,487 orthologous groups were identified by compared with 150 genomes [17]. This might reflect that not all genes were expressed at the sampling point.

### Polymorphism rate in Porites

Since total RNA was extracted from a single *Porites* colony, it is possible to estimate the polymorphism rate of *P. australiensis* within an individual. High quality Illumina reads were re-mapped against the reference *Porites* contigs using BWA software [42], and high quality SNPs and small indels (∼5 bp) within the *Porites* colony were identified using SAMtools [43]. Among the 44,569,031 nucleotides of the 26,658 *Porites* contigs, 425,728 SNPs and 11,788 small indels (total 20,213 bp) were detected. Taken together, the polymorphism rate between haplotypes, including small indels (less than 5 bp) and SNPs in the exons of *Porites* transcripts is estimated to be 1.0%, which is slightly higher than that observed in other cnidarian genomes (*A. digitifera* [0.4%, Shinzato and Mungpakdee, unpublished data], *Nematostella* [0.65%, 8], and *Hydra* [0.69%, 9].

### Symbiont type

The genus *Symbiodinium* displays tremendous taxonomic diversity with nine divergent lineages. Clades A–I have been described in *Symbiodinium* based on nuclear ribosomal DNA (rDNA) and chloroplast 23S rDNA [51]. Each clade contains multiple genetic types, often resolved using the internal transcribed spacer 2 (ITS-2) regions [44], [52]. We failed to detect assembled sequences of nuclear rDNA or chloroplast 23S rDNA in our dataset. Accordingly, the ITS2 region-containing sequence reads were identified and assembled, and then we managed to reconstruct an ITS-2 region (341 bp) of the symbiotic *Symbiodinium*. BLAST homology searches against the NCBI NT database revealed that this sequence shows high similarity (340/341 bp identity) with clade C *Symbiodinium* sp. C15 (GenBank: JN558044). We also checked the sequence variation within the ITS2 region by BWA mapping and SAMtools, but no variation was detected (data not shown), indicating that the C15 type, a type common in *Porites* species across the Pacific [53], is the major *Symbiodinium* type in the *P. australiensis* colony used in this study.

### Amino acid biosynthesis pathways in Porites holobiont

Bacteria, plants, and many fungi are able to synthesize all of the 20 amino acids commonly found in proteins, whereas all animals studied to date either lack the ability to synthesize one or more of these amino acids, or else they are unable to synthesize quantities sufficient to meet their metabolic needs. These amino acids are termed “essential” and must be obtained from the diet. For vertebrates, eight or more amino acids are essential; threonine, valine, methionine, leucine, isoleucine, phenylalanine, lysine, and tryptophan are required by all vertebrates, while arginine and/or histidine are also essential in some cases [54]. Although amino acid biosynthesis occurs via conserved pathways and is relatively well-characterized [55], amino acid exchange in cnidarian photoautotrophic symbioses is poorly understood. The issue of amino acid requirements is complicated by the presence of symbiotic algae [56], [57]. For example, examination of the amino acid biosynthetic capacity of the sea anemone *Aiptasia pulchella* – a facultative host of *Symbiodinium* sp. – suggested that seven amino acids (histidine, isoleucine, leucine, lysine, phenylalanine, tyrosine, and valine) are synthesized by the symbiotic algae and translocated to the sea anemone, and that methionine and threonine are likely to be synthesized by *A. pulchella* itself [57]. A genome-wide survey of amino acid biosynthetic pathway components in *A. digitifera* revealed that *Acropora* corals may be able to synthesize ten non-essential amino acids, but not cysteine [10], however amino acid metabolism in coral holobionts is still unclear (Figure? 4A). We reconstructed the amino acid biosynthetic pathways in the *Porites* holobiont based on KEGG IDs (Figure? 4B, C). In this study we took amino acid biosynthetic pathways missing one or two enzymes into consideration. Most of enzymes involved in amino acid biosynthetic pathways were detected in the transcriptomes of *Porites* and *Symbiodinium*, while four enzymes cannot be detected in the lysine anabolic pathway (Figure? 3C).

Interestingly many enzymes involved in essential amino acid biosynthesis are detected only in *Symbiodinium* contigs (Figure? 3B, Figure? 3C left side). Overrepresentation of “transport” GO terms in *Symbiodinium* contigs (Figure S1) implies that *Symbiodinium* transport essential amino acids to host cells. In contrast, enzymes for non-essential amino acid pathways were detected in both *Symbiodinium* and *Porites* (Figure? 3B, Figure? 3C, right side). Glutamic acid, glutamine, aspartic acid, and alanine are probably produced in both *Porites* and *Symbiodinium*. According our prediction, asparagine is synthesized in *Symbiodinium* while cysteine is synthesized in *Porites* (Figure? 3). We have shown previously that *Acropora* corals lack an essential enzyme for cysteine biosynthesis, cystatione ß-synthase [10], however, *Porites* seems to posses it (Figure? 3C), indicating that it does not depend upon *Symbiodinium* for cysteine biosynthesis and might account for its greater resilience to environmental stresses. An interesting example is the methionine biosynthesis pathway. Half of the enzymes reside in the *Porites* and *Symbiodinium* contigs, respectively, suggesting that methionine might be produced by intimate cooperation between host and symbiont in coral holobionts. While transcriptome sequencing was based upon a single RNA-seq library in this study, we were able to recover a surprisingly large gene repertoire for amino acid biosynthesis. Our data appear to provide the first direct molecular evidence of complementarity and syntrophy between coral hosts and their symbionts in amino acid metabolism.

## Conclusions

We sequenced a *Porites australiensis* holobiont and demonstrated that decoded genomic data of an *Acropora* coral [10] and a *Symbiodinium minutum* [17] greatly assisted the characterization of assembled contigs from a mixture of RNAs from different organisms. In this study we were able distinguish about 70% of contigs as host or symbiont. The assembled sequences contain a wide variety of genetic information from both the coral and its symbiont, *Symbiodinium*, including genes for most enzymes in all amino acid biosynthetic pathways. When genome sequences of *P. australiensis* and its symbiotic *Symbiodinium* are available, the annotation could be further improved. The *Porites* holobiont transcriptome dataset allows us to utilize *Porites australiensis*, an abundant coral species from the Indo-Pacific, to reveal molecular mechanisms of coral symbiosis and coral stress responses. Our approach provides an opportunity to simultaneously analyze coral-symbiont interactions on transcriptomic level. Furthermore, analyzing the molecular bases of *Porites* calcification may enable researchers to improve the accuracy of future climate change prediction.

## Supporting Information

Figure S1 **Analysis of GO term enrichment of the “biological process” category for** ***Porites*** **and** ***Symbiodinium*** **contigs.** GO terms containing at least 100 sequences are shown. The Y-axis represents proportions of contigs in each category of GO-assigned contigs (*Porites*: 9806, *Symbiodinium*: 9147). Blue bars indicate *Porites* contigs. Red bars indicate *Symbiodinium* contigs.
(PDF)

Figure S2 **Analysis of GO term enrichment of the “cellular component” category for** ***Porites*** **and** ***Symbiodinium*** **contigs.** GO terms containing at least 100 sequences are shown. The Y-axis represents proportions of contigs in each category of GO-assigned contigs (*Porites*: 9806, *Symbiodinium*: 9147). Blue bars indicate *Porites* contigs. Red bars indicate *Symbiodinium* contigs.
(PDF)

Figure S3 **Analysis of GO term enrichment of the “molecular function” category for** ***Porites*** **and** ***Symbiodinium*** **contigs.** GO terms containing at least 100 sequences are shown. The Y-axis represents proportions of contigs in each category of GO-assigned contigs (*Porites*: 9806, *Symbiodinium*: 9147). Blue bars indicate *Porites* contigs. Red bars indicate *Symbiodinium* contigs.
(PDF)

Table S1 **Summary of the sequencing data.**
(PDF)

Table S2 **Summary of published anthozoan transcriptome assemblies.** References. 1. Moya A, Huisman L, Ball EE, Hayward DC, Grasso LC, Chua CM, Woo HN, Gattuso JP, Foret S, Miller DJ: Whole transcriptome analysis of the coral *Acropora millepora* reveals complex responses to CO_2_-driven acidification during the initiation of calcification. Mol Ecol 2012, 21:2440–2454, 2. Polato NR, Vera JC, Baums IB: Gene discovery in the threatened elkhorn coral: 454 sequencing of the *Acropora palmata* transcriptome. PLoS One 2011, 6:e28634. 3. Traylor-Knowles N, Granger BR, Lubinski TJ, Parikh JR, Garamszegi S, Xia Y, Marto JA, Kaufman L, Finnerty JR: Production of a reference transcriptome and transcriptomic database (PocilloporaBase) for the cauliflower coral, *Pocillopora damicornis*. BMC Genomics 2011, 12:585. 4. Lehnert EM, Burriesci MS, Pringle JR: Developing the anemone *Aiptasia* as a tractable model for cnidarian-dinoflagellate symbiosis: the transcriptome of aposymbiotic *A. pallida*. BMC Genomics 2012, 13:271.
(PDF)

Table S3 **Results of nucleotide sequence alignment of the assembled sequences with different e-value settings of BLASTN against** ***Acropora digitifera*** **and** ***Symbiodinium minutum*** **genome sequences.** We selected e-value cut-off as 1e^−4^ in this study as the number of *Porites* contigs is the largest.
(PDF)

Table S4 **Comparison of the number of genes with transcription factor-related domains of** ***Porites australiensis*** **,** ***Acropora digitifera*** **,** ***Nematostella vectensis*** **and** ***Hydra magnipapillata*** **.**
(PDF)

Table S5 **Comparison of the number of genes with signaling molecule-related domains of** ***Porites australiensis*** **,** ***Acropora digitifera*** **,** ***Nematostella vectensis*** **and** ***Hydra magnipapillata*** **.**
(PDF)

File S1 **List of** ***Porites*** **contigs (tab-separated text; contig names and NCBI accessions).**
(ZIP)

File S2 **List of** ***Symbiodinium*** **contigs (tab-separated text; contig names and NCBI accessions).**
(ZIP)

File S3 **Annotation files for all contigs (tab-separated text; contig name, NCBI accession, Swiss-Prot blast result, Pfam ID, KEGG ID).**
(ZIP)

## References

[1] Yellowlees D, Rees TA, Leggat W (2008) Metabolic interactions between algal symbionts and invertebrate hosts. Plant Cell Env 31: 679–694 Available: http://www.ncbi.nlm.nih.gov/pubmed/18315536. 18315536 10.1111/j.1365-3040.2008.01802.x

[2] Hohman TC, McNeil PL, Muscatine L (1982) Phagosome-lysosome fusion inhibited by algal symbionts of Hydra viridis. J? Cell Biol 94: 56–63 Available: http://www.ncbi.nlm.nih.gov/pubmed/7119017. 7119017 10.1083/jcb.94.1.56 PMC2112179

[3] Carpenter KE, Abrar M, Aeby G, Aronson RB, Banks S, et al (2008) One-third of reef-building corals face elevated extinction risk from climate change and local impacts. Science (80-) 321: 560–563 Available: http://www.ncbi.nlm.nih.gov/entrez/query.fcgi?cmd=Retrieve&db=PubMed&dopt=Citation&list_uids=18653892. 10.1126/science.1159196 18653892

[4] Hoegh-Guldberg O, Mumby PJ, Hooten AJ, Steneck RS, Greenfield P, et al (2007) Coral reefs under rapid climate change and ocean acidification. Science (80-) 318: 1737–1742 Available: http://www.ncbi.nlm.nih.gov/pubmed/18079392. 10.1126/science.1152509 18079392

[5] Hughes TP, Baird AH, Bellwood DR, Card M, Connolly SR, et al (2003) Climate change, human impacts, and the resilience of coral reefs. Science (80-) 301: 929–933 Available: http://www.ncbi.nlm.nih.gov/entrez/query.fcgi?cmd=Retrieve&db=PubMed&dopt=Citation&list_uids=12920289. 10.1126/science.1085046 12920289

[6] Bourne DG, Garren M, Work TM, Rosenberg E, Smith GW, et al (2009) Microbial disease and the coral holobiont. Trends Microbiol 17: 554–562 Available: http://www.ncbi.nlm.nih.gov/pubmed/19822428. 19822428 10.1016/j.tim.2009.09.004

[7] Weis VM (2010) The susceptibility and resilience of corals to thermal stress: adaptation, acclimatization or both? Mol Ecol 19: 1515–1517 Available: http://www.ncbi.nlm.nih.gov/entrez/query.fcgi?cmd=Retrieve&db=PubMed&dopt=Citation&list_uids=20456235. 20456235 10.1111/j.1365-294X.2010.04575.x

[8] Putnam NH, Srivastava M, Hellsten U, Dirks B, Chapman J, et al (2007) Sea anemone genome reveals ancestral eumetazoan gene repertoire and genomic organization. Science (80-) 317: 86–94 Available: http://www.ncbi.nlm.nih.gov/entrez/query.fcgi?cmd=Retrieve&db=PubMed&dopt=Citation&list_uids=17615350. 10.1126/science.1139158 17615350

[9] Chapman JA, Kirkness EF, Simakov O, Hampson SE, Mitros T, et al (2010) The dynamic genome of Hydra. Nature 464: 592–596 Available: http://www.ncbi.nlm.nih.gov/entrez/query.fcgi?cmd=Retrieve&db=PubMed&dopt=Citation&list_uids=20228792. 20228792 10.1038/nature08830 PMC4479502

[10] Shinzato C, Shoguchi E, Kawashima T, Hamada M, Hisata K, et al (2011) Using the Acropora digitifera genome to understand coral responses to environmental change. Nature 476: 320–323 Available: http://www.ncbi.nlm.nih.gov/entrez/query.fcgi?cmd=Retrieve&db=PubMed&dopt=Citation&list_uids=21785439. 21785439 10.1038/nature10249

[11] Meyer E, Aglyamova G V, Wang S, Buchanan-Carter J, Abrego D, et al (2009) Sequencing and de novo analysis of a coral larval transcriptome using 454 GSFlx. BMC Genomics 10: 219 Available: http://www.ncbi.nlm.nih.gov/pubmed/19435504. 19435504 10.1186/1471-2164-10-219 PMC2689275

[12] Moya A, Huisman L, Ball EE, Hayward DC, Grasso LC, et al (2012) Whole transcriptome analysis of the coral Acropora millepora reveals complex responses to CO(2)-driven acidification during the initiation of calcification. Mol Ecol 21: 2440–2454 Available: http://www.ncbi.nlm.nih.gov/entrez/query.fcgi?cmd=Retrieve&db=PubMed&dopt=Citation&list_uids=22490231. 22490231 10.1111/j.1365-294X.2012.05554.x

[13] Polato NR, Vera JC, Baums IB (2011) Gene discovery in the threatened elkhorn coral: 454 sequencing of the Acropora palmata transcriptome. PLoS One 6: e28634 Available: http://www.ncbi.nlm.nih.gov/pubmed/22216101. 22216101 10.1371/journal.pone.0028634 PMC3247206

[14] Traylor-Knowles N, Granger BR, Lubinski TJ, Parikh JR, Garamszegi S, et al (2011) Production of a reference transcriptome and transcriptomic database (PocilloporaBase) for the cauliflower coral, Pocillopora damicornis. BMC Genomics 12: 585 Available: http://www.ncbi.nlm.nih.gov/pubmed/22126435. 22126435 10.1186/1471-2164-12-585 PMC3339375

[15] Lehnert EM, Burriesci MS, Pringle JR (2012) Developing the anemone Aiptasia as a tractable model for cnidarian-dinoflagellate symbiosis: the transcriptome of aposymbiotic A. pallida. BMC Genomics 13: 271 Available: http://www.ncbi.nlm.nih.gov/pubmed/22726260. 22726260 10.1186/1471-2164-13-271 PMC3427133

[16] Bayer T, Aranda M, Sunagawa S, Yum LK, Desalvo MK, et al (2012) Symbiodinium transcriptomes: genome insights into the dinoflagellate symbionts of reef-building corals. PLoS One 7: e35269 Available: http://www.ncbi.nlm.nih.gov/entrez/query.fcgi?cmd=Retrieve&db=PubMed&dopt=Citation&list_uids=22529998. Accessed 29 November 2012. 22529998 10.1371/journal.pone.0035269 PMC3329448

[17] Shoguchi E, Shinzato C, Kawashima T, Gyoja F, Mungpakdee S, et al (2013) Draft Assembly of the Symbiodinium minutum Nuclear Genome Reveals Dinoflagellate Gene Structure. Curr Biol Available: http://www.ncbi.nlm.nih.gov/pubmed/23850284. 10.1016/j.cub.2013.05.062 23850284

[18] Appeltans W Boxshall GA, De Broyer C, de Voogd NJ, Gordon DP, Hoeksema BW, Horton T, Kennedy M, Mees J, Poore GCB, Read G, Stöhr S, Walter TC, Costello MJ BP (2012) World Register of Marine Species.

[19] LaJeunesse TC, Thornhill DJ, Cox EF, Stanton FG, Fitt WK, et al (2004) High diversity and host specificity observed among symbiotic dinoflagellates in reef coral communities from Hawaii. Coral Reefs 23: 596–603 Available: <Go to ISI>://000226095100021.

[20] Loya Y, Sakai K, Yamazato K, Nakano Y, Sambali H, et al (2001) Coral bleaching: the winners and the losers. Ecol Lett 4: 122–131 Available: <Go to ISI>://000167731300005.

[21] Fitt WK, Gates RD, Hoegh-Guldberg O, Bythell JC, Jatkar A, et al (2009) Response of two species of Indo-Pacific corals, Porites cylindrica and Stylophora pistillata, to short-term thermal stress: The host does matter in determining the tolerance of corals to bleaching. J? Exp Mar Bio Ecol 373: 102–110 Available: <Go to ISI>://000266897400004.

[22] Anthony KRN, Kline DI, Diaz-Pulido G, Dove S, Hoegh-Guldberg O (2008) Ocean acidification causes bleaching and productivity loss in coral reef builders. Proc Natl Acad Sci U S A 105: 17442–17446 Available: <Go to ISI>://000260981800051. 18988740 10.1073/pnas.0804478105 PMC2580748

[23] Iguchi A, Ozaki S, Nakamura T, Inoue M, Tanaka Y, et al (2012) Effects of acidified seawater on coral calcification and symbiotic algae on the massive coral Porites australiensis. Mar Environ Res 73: 32–36 Available: <Go to ISI>://000300818900005. 22115919 10.1016/j.marenvres.2011.10.008

[24] Ohde S, Hossain MMM (2004) Effect of CaCO3 (aragonite) saturation state of seawater on calcification of Porites coral. Geochem? J 38: 613–621 Available: <Go to ISI>://000226353600012.

[25] Beck JW, Edwards RL, Ito E, Taylor FW, Recy J, et al (1992) Sea-Surface Temperature from Coral Skeletal Strontium Calcium Ratios. Science (80-) 257:: 644–647 Available: <Go to ISI>://A1992JF85200028. 10.1126/science.257.5070.644 17740731

[26] Gagan MK, Ayliffe LK, Beck JW, Cole JE, Druffel ERM, et al (2000) New views of tropical paleoclimates from corals. Quat Sci Rev 19: 45–64 Available: <Go to ISI>://000084425500006.

[27] Inoue M, Tanimizu M (2008) Anthropogenic lead inputs to the western Pacific during the 20th century. Sci Total Environ 406: 123–130 Available: <Go to ISI>://000260941400012. 18775557 10.1016/j.scitotenv.2008.07.032

[28] Shen GT, Boyle EA, Lea DW (1987) Cadmium in Corals as a Tracer of Historical Upwelling and Industrial Fallout. Nature 328: 794–796 Available: <Go to ISI>://A1987J762900054.

[29] Cobb KM, Charles CD, Cheng H, Edwards RL (2003) El Nino/Southern Oscillation and tropical Pacific climate during the last millennium. Nature 424: 271–276 Available: http://www.ncbi.nlm.nih.gov/pubmed/12867972. 12867972 10.1038/nature01779

[30] Solomon S, Intergovernmental Panel on Climate Change., Intergovernmental Panel on Climate Change. Working Group I. (2007) Climate change 2007: the physical science basis: contribution of Working Group I to the Fourth Assessment Report of the Intergovernmental Panel on Climate Change. Cambridge; New York: Cambridge University Press. Available: http://www.loc.gov/catdir/enhancements/fy0806/2007282362-d.html.

[31] Cox MP, Peterson DA, Biggs PJ (2010) SolexaQA: At-a-glance quality assessment of Illumina second-generation sequencing data. BMC Bioinformatics 11: 485 Available: http://www.ncbi.nlm.nih.gov/pubmed/20875133. 20875133 10.1186/1471-2105-11-485 PMC2956736

[32] Smeds L, Kunstner A (2011) ConDeTri–a content dependent read trimmer for Illumina data. PLoS One 6: e26314 Available: http://www.ncbi.nlm.nih.gov/pubmed/22039460. 22039460 10.1371/journal.pone.0026314 PMC3198461

[33] Zerbino DR, Birney E (2008) Velvet: algorithms for de novo short read assembly using de Bruijn graphs. Genome Res 18: 821–829 Available: http://www.ncbi.nlm.nih.gov/entrez/query.fcgi?cmd=Retrieve&db=PubMed&dopt=Citation&list_uids=18349386. 18349386 10.1101/gr.074492.107 PMC2336801

[34] Schulz MH, Zerbino DR, Vingron M, Birney E (2012) Oases: robust de novo RNA-seq assembly across the dynamic range of expression levels. Bioinformatics 28: 1086–1092 Available: http://www.ncbi.nlm.nih.gov/entrez/query.fcgi?cmd=Retrieve&db=PubMed&dopt=Citation&list_uids=22368243. 22368243 10.1093/bioinformatics/bts094 PMC3324515

[35] Li W, Godzik A (2006) Cd-hit: a fast program for clustering and comparing large sets of protein or nucleotide sequences. Bioinformatics 22: 1658–1659 Available: http://www.ncbi.nlm.nih.gov/entrez/query.fcgi?cmd=Retrieve&db=PubMed&dopt=Citation&list_uids=16731699. 16731699 10.1093/bioinformatics/btl158

[36] Consortium U (2011) Ongoing and future developments at the Universal Protein Resource. Nucleic Acids Res 39: D214–9 Available: http://www.ncbi.nlm.nih.gov/pubmed/21051339. 21051339 10.1093/nar/gkq1020 PMC3013648

[37] Ashburner M, Ball CA, Blake JA, Botstein D, Butler H, et al (2000) Gene ontology: tool for the unification of biology. The Gene Ontology Consortium. Nat Genet 25: 25–29 Available: http://www.ncbi.nlm.nih.gov/pubmed/10802651. 10802651 10.1038/75556 PMC3037419

[38] Moriya Y, Itoh M, Okuda S, Yoshizawa AC, Kanehisa M (2007) KAAS: an automatic genome annotation and pathway reconstruction server. Nucleic Acids Res 35: W182–5 Available: http://www.ncbi.nlm.nih.gov/pubmed/17526522. 17526522 10.1093/nar/gkm321 PMC1933193

[39] Finn RD, Mistry J, Schuster-Bockler B, Griffiths-Jones S, Hollich V, et al (2006) Pfam: clans, web tools and services. Nucleic Acids Res 34: D247–51 Available: http://www.ncbi.nlm.nih.gov/pubmed/16381856. 16381856 10.1093/nar/gkj149 PMC1347511

[40] Eddy SR (1998) Profile hidden Markov models. Bioinformatics 14: 755–763 Available: http://www.ncbi.nlm.nih.gov/pubmed/9918945. 9918945 10.1093/bioinformatics/14.9.755

[41] Kawashima T, Kawashima S, Tanaka C, Murai M, Yoneda M, et al (2009) Domain shuffling and the evolution of vertebrates. Genome Res 19: 1393–1403 Available: http://www.ncbi.nlm.nih.gov/pubmed/19443856. 19443856 10.1101/gr.087072.108 PMC2720177

[42] Li H, Durbin R (2009) Fast and accurate short read alignment with Burrows-Wheeler transform. Bioinformatics 25: 1754–1760 Available: http://www.ncbi.nlm.nih.gov/pubmed/19451168. 19451168 10.1093/bioinformatics/btp324 PMC2705234

[43] Li H, Handsaker B, Wysoker A, Fennell T, Ruan J, et al (2009) The Sequence Alignment/Map format and SAMtools. Bioinformatics 25: 2078–2079 Available: http://www.ncbi.nlm.nih.gov/pubmed/19505943. 19505943 10.1093/bioinformatics/btp352 PMC2723002

[44] Pochon X, Garcia-Cuetos L, Baker AC, Castella E, Pawlowski J (2007) One-year survey of a single Micronesian reef reveals extraordinarily rich diversity of Symbiodinium types in soritid foraminifera. Coral Reefs 26: 867–882 Available: <Go to ISI>://000251579300016.

[45] Gordon D, Abajian C, Green P (1998) Consed: a graphical tool for sequence finishing. Genome Res 8: 195–202 Available: http://www.ncbi.nlm.nih.gov/pubmed/9521923. 9521923 10.1101/gr.8.3.195

[46] Shinzato C, Iguchi A, Hayward DC, Technau U, Ball EE, et al (2008) Sox genes in the coral Acropora millepora: divergent expression patterns reflect differences in developmental mechanisms within the Anthozoa. BMC Evol Biol 8: 311 Available: http://www.ncbi.nlm.nih.gov/entrez/query.fcgi?cmd=Retrieve&db=PubMed&dopt=Citation&list_uids=19014479. 19014479 10.1186/1471-2148-8-311 PMC2613919

[47] De Jong DM, Hislop NR, Hayward DC, Reece-Hoyes JS, Pontynen PC, et al (2006) Components of both major axial patterning systems of the Bilateria are differentially expressed along the primary axis of a “radiate” animal, the anthozoan cnidarian Acropora millepora. Dev Biol 298: 632–643 Available: <Go to ISI>://000241180300024. 16952346 10.1016/j.ydbio.2006.07.034

[48] Kusserow A, Pang K, Sturm C, Hrouda M, Lentfer J, et al (2005) Unexpected complexity of the Wnt gene family in a sea anemone. Nature 433: 156–160 Available: http://www.ncbi.nlm.nih.gov/pubmed/15650739. 15650739 10.1038/nature03158

[49] Dubuc TQ, Ryan JF, Shinzato C, Satoh N, Martindale MQ (2012) Coral Comparative Genomics Reveal Expanded Hox Cluster in the Cnidarian-Bilaterian Ancestor. Integr Comp Biol Available: http://www.ncbi.nlm.nih.gov/entrez/query.fcgi?cmd=Retrieve&db=PubMed&dopt=Citation&list_uids=22767488. 10.1093/icb/ics098 PMC4817585 22767488

[50] Simionato E, Ledent V, Richards G, Thomas-Chollier M, Kerner P, et al (2007) Origin and diversification of the basic helix-loop-helix gene family in metazoans: insights from comparative genomics. BMC Evol Biol 7 Available: <Go to ISI>://000244937200001. 10.1186/1471-2148-7-33 PMC1828162 17335570

[51] Pochon X, Gates RD (2010) A new Symbiodinium clade (Dinophyceae) from soritid foraminifera in Hawai'i. Mol Phylogenet Evol 56: 492–497 Available: <Go to ISI>://000278589500047. 20371383 10.1016/j.ympev.2010.03.040

[52] Stat M, Bird CE, Pochon X, Chasqui L, Chauka LJ, et al (2011) Variation in Symbiodinium ITS2 sequence assemblages among coral colonies. PLoS One 6: e15854 Available: http://www.ncbi.nlm.nih.gov/pubmed/21246044. 21246044 10.1371/journal.pone.0015854 PMC3016399

[53] LaJeunesse TC, Loh WKW, van Woesik R, Hoegh-Guldberg O, Schmidt GW, et al (2003) Low symbiont diversity in southern Great Barrier Reef corals, relative to those of the Caribbean. Limnol Oceanogr 48: 2046–2054 Available: <Go to ISI>://000185433700030.

[54] Furst P, Stehle P (2004) What are the essential elements needed for the determination of amino acid requirements in humans? J? Nutr 134: 1558S–1565S Available: http://www.ncbi.nlm.nih.gov/pubmed/15173430. 15173430 10.1093/jn/134.6.1558S

[55] Shigenobu S, Watanabe H, Hattori M, Sakaki Y, Ishikawa H (2000) Genome sequence of the endocellular bacterial symbiont of aphids Buchnera sp. APS. Nature 407: 81–86 Available: http://www.ncbi.nlm.nih.gov/pubmed/10993077. 10993077 10.1038/35024074

[56] Swanson R, Hoegh-Guldberg O (1998) Amino acid synthesis in the symbiotic sea anemone Aiptasia pulchella. Mar Biol 131: 83–93 Available: <Go to ISI>://000073613500010.

[57] Wang JT, Douglas AE (1999) Essential amino acid synthesis and nitrogen recycling in an alga-invertebrate symbiosis. Mar Biol 135: 219–222 Available: <Go to ISI>://000083839200002.

