# Supplementary figures and images for: A Snapshot of a Coral “Holobiont”: A Transcriptome Assembly of the Scleractinian Coral, Porites, Captures a Wide Variety of Genes from Both the Host and Symbiotic Zooxanthellae
Source: PLoS One. 2014 Jan 15;9(1):e85182. doi: 10.1371/journal.pone.0085182 (PMC3893191; doi:10.1371/journal.pone.0085182)

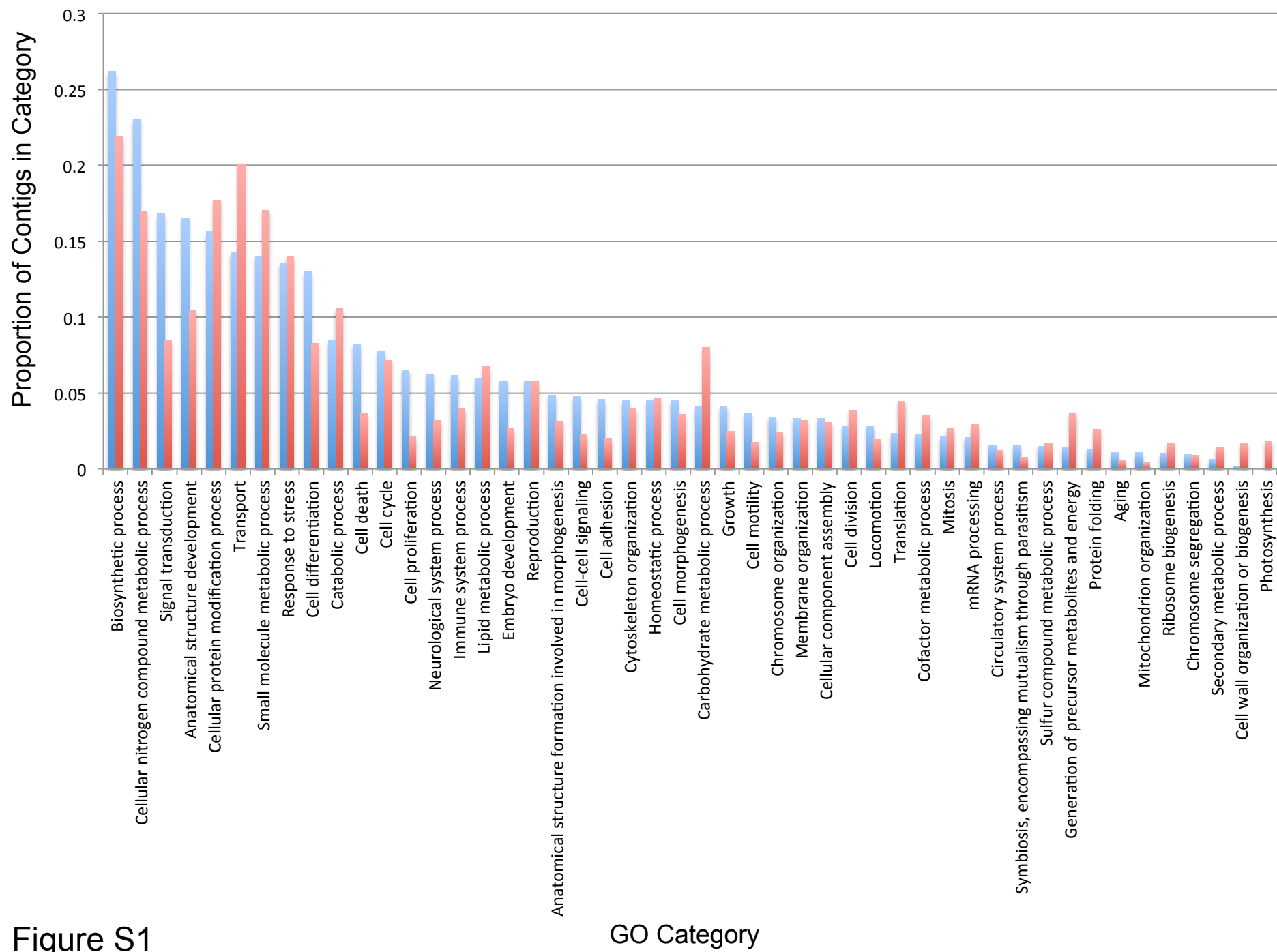

Figure S1

Supplement: Figure S1 — Analysis of GO term enrichment of the “biological process” category for Porites and Symbiodinium contigs. GO terms containing at least 100 sequences are shown. The Y-axis represents proportions of contigs in each category of GO-assigned contigs (Porites: 9806, Symbiodinium: 9147). Blue bars indicate Porites contigs. Red bars indicate Symbiodinium contigs. (PDF) [file pone.0085182.s001.pdf]

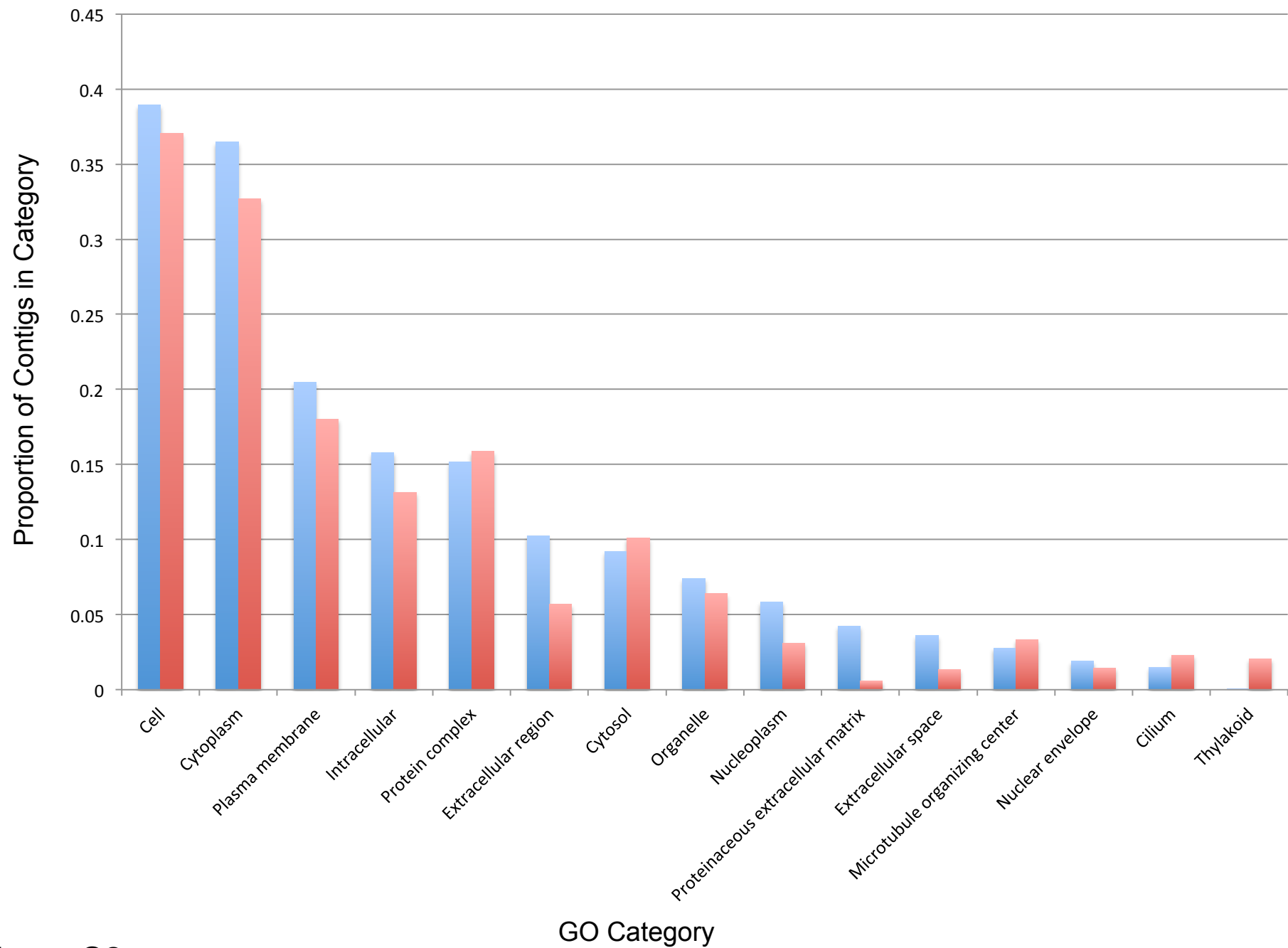

Figure S2

Supplement: Figure S2 — Analysis of GO term enrichment of the “cellular component” category for Porites and Symbiodinium contigs. GO terms containing at least 100 sequences are shown. The Y-axis represents proportions of contigs in each category of GO-assigned contigs (Porites: 9806, Symbiodinium: 9147). Blue bars indicate Porites contigs. Red bars indicate Symbiodinium contigs. (PDF) [file pone.0085182.s002.pdf]
