# Supplementary material for: A Snapshot of a Coral “Holobiont”: A Transcriptome Assembly of the Scleractinian Coral, Porites, Captures a Wide Variety of Genes from Both the Host and Symbiotic Zooxanthellae
Source: PLoS One. 2014 Jan 15;9(1):e85182. doi: 10.1371/journal.pone.0085182 (PMC3893191; doi:10.1371/journal.pone.0085182)
Supplement: Table S1 — Summary of the sequencing data. (PDF) [file pone.0085182.s004.pdf]

| | |
|---|---|
| Number of raw read pair | 70910785 |
| Average length (bp) | 101 |
| Total basepair | 14323978570 |
| Number of trimmed read pair | 44967583 |
| Average length (bp) | 100.6 |
| Total basepair | 9043943642 |
