# Supplementary material for: A Snapshot of a Coral “Holobiont”: A Transcriptome Assembly of the Scleractinian Coral, Porites, Captures a Wide Variety of Genes from Both the Host and Symbiotic Zooxanthellae
Source: PLoS One. 2014 Jan 15;9(1):e85182. doi: 10.1371/journal.pone.0085182 (PMC3893191; doi:10.1371/journal.pone.0085182)
Supplement: Table S2 — Summary of published anthozoan transcriptome assemblies. References. 1. Moya A, Huisman L, Ball EE, Hayward DC, Grasso LC, Chua CM, Woo HN, Gattuso JP, Foret S, Miller DJ: Whole transcriptome analysis of the coral Acropora millepora reveals complex responses to CO2-driven acidification during the initiation of calcification. Mol Ecol 2012, 21:2440–2454, 2. Polato NR, Vera JC, Baums IB: Gene discovery in the threatened elkhorn coral: 454 sequencing of the Acropora palmata transcriptome. PLoS One 2011, 6:e28634. 3. Traylor-Knowles N, Granger BR, Lubinski TJ, Parikh JR, Garamszegi S, Xia Y, Marto JA, Kaufman L, Finnerty JR: Production of a reference transcriptome and transcriptomic database (PocilloporaBase) for the cauliflower coral, Pocillopora damicornis. BMC Genomics 2011, 12:585. 4. Lehnert EM, Burriesci MS, Pringle JR: Developing the anemone Aiptasia as a tractable model for cnidarian-dinoflagellate symbiosis: the transcriptome of aposymbiotic A. pallida. BMC Genomics 2012, 13:271. (PDF) [file pone.0085182.s005.pdf]

Species	Number of contigs	Total size (Mbp)	Average length (bp)	N50 size	Longest contig (bp)	Sequencing platform	Reference
Acropora millepora	56,260	77	1,367	2,023	35,256	Sanger, 454, Illumina	1
Acropora palmata	88,020	64	725	802	9,066	Sanger, 454	2
Pocillopora damicornis	70,786	59	836	976	10,512	454	3
Aiptasia pallida	58,018	45	770	1,185	13,061	Illumina	4
