# Supplementary material for: A Snapshot of a Coral “Holobiont”: A Transcriptome Assembly of the Scleractinian Coral, Porites, Captures a Wide Variety of Genes from Both the Host and Symbiotic Zooxanthellae
Source: PLoS One. 2014 Jan 15;9(1):e85182. doi: 10.1371/journal.pone.0085182 (PMC3893191; doi:10.1371/journal.pone.0085182)
Supplement: Table S3 — Results of nucleotide sequence alignment of the assembled sequences with different e-value settings of BLASTN against Acropora digitifera and Symbiodinium minutum genome sequences. We selected e-value cut-off as 1e−4 in this study as the number of Porites contigs is the largest. (PDF) [file pone.0085182.s006.pdf]

**E-value**	***A. digitiefra* hit**	***S. minutum* hit**	**Both *A. digitifera* and *S. minutum* hit**	***A. digitifera* hit only (*Porites* contigs)**	***S.minutum* hit only (*Symbiodinium* contig)**
$1e^{-1}$	39419	48439	21250	18169	27189
$1e^{-2}$	33552	35754	8388	25164	27366
$1e^{-3}$	31759	32235	5226	26533	27009
**$1e^{-4}$**	**30446**	**30415**	**3788**	**26658**	**26627**
$1e^{-5}$	29321	29203	3044	26277	26159
$1e^{-10}$	25504	24334	1503	24001	22831
