# Supplementary material for: A Snapshot of a Coral “Holobiont”: A Transcriptome Assembly of the Scleractinian Coral, Porites, Captures a Wide Variety of Genes from Both the Host and Symbiotic Zooxanthellae
Source: PLoS One. 2014 Jan 15;9(1):e85182. doi: 10.1371/journal.pone.0085182 (PMC3893191; doi:10.1371/journal.pone.0085182)
Supplement: Table S4 — Comparison of the number of genes with transcription factor-related domains of Porites australiensis , Acropora digitifera , Nematostella vectensis and Hydra magnipapillata . (PDF) [file pone.0085182.s007.pdf]

Domain name	Accession	Description	*Porites*	*Acropora*	*Nematostella*	*Hydra*
HLH	PF00010	Helix-loop-helix DNA-binding domain	48	52	72	32
Homeobox	PF00046	Homeobox domain	89	97	155	43
Hormone_rece p	PF00104	Ligand-binding domain of nuclear hormone receptor	12	9	21	7
Pou	PF00157	Pou domain - N-terminal to homeobox domain	4	4	6	3
bZIP_1	PF00170	bZIP transcription factor	19	24	38	24
Ets	PF00178	Ets-domain	15	12	16	9
Fork_head	PF00250	Fork head domain	21	22	34	15
PAX	PF00292	'Paired box' domain	8	8	9	27
SRF-TF	PF00319	SRF-type transcription factor (DNA-binding and dimerisation domain)	2	1	4	2
GATA	PF00320	GATA zinc finger	2	5	5	5
HMG_box	PF00505	HMG (high mobility group) box	24	26	35	33
RHD	PF00554	Rel homology domain (RHD)	2	2	3	1
DM	PF00751	DM DNA binding domain	3	8	12	6
Runt	PF00853	Runt domain	0	1	1	1
P53	PF00870	P53 DNA-binding domain	3	3	3	2
T-box	PF00907	T-box	8	10	16	7
ARID	PF01388	ARID/BRIGHT DNA binding domain	7	5	6	7
Basic	PF01586	Myogenic Basic domain	0	0	0	0
AT_hook	PF02178	AT hook motif	1	0	0	0
CUT	PF02376	CUT domain	3	1	2	0
TF_AP-2	PF03299	Transcription factor AP-2	2	2	1	1
TF_Otx	PF03529	Otx1 transcription factor	0	0	0	0
GCM	PF03615	GCM motif protein	1	1	2	0
OAR	PF03826	OAR domain	7	5	4	0
Prox1	PF05044	Homeobox prospero-like protein (PROX1)	0	0	0	0
SIM_C	PF06621	Single-minded protein C-terminus	0	0	0	0
Hairy_orange	PF07527	Hairy Orange	6	7	6	0
P53_tetramer	PF07710	P53 tetramerisation motif	1	1	1	0
bZIP_2	PF07716	Basic region leucine zipper	15	17	34	20
zf-C2H2	PF00096	Zinc finger, C2H2 type	167	88	209	106
zf-C4	PF00105	Zinc finger, C4 type (two domains)	11	12	19	8
zf-C2HC	PF01530	Zinc finger, C2HC type	4	4	4	2
SCAN	PF02023	SCAN domain	1	4	0	0
