# Supplementary material for: A Snapshot of a Coral “Holobiont”: A Transcriptome Assembly of the Scleractinian Coral, Porites, Captures a Wide Variety of Genes from Both the Host and Symbiotic Zooxanthellae
Source: PLoS One. 2014 Jan 15;9(1):e85182. doi: 10.1371/journal.pone.0085182 (PMC3893191; doi:10.1371/journal.pone.0085182)
Supplement: Table S5 — Comparison of the number of genes with signaling molecule-related domains of Porites australiensis , Acropora digitifera , Nematostella vectensis and Hydra magnipapillata . (PDF) [file pone.0085182.s008.pdf]

**Domain name**	**Accession**	**Description**	***Porites***	***Acropora***	***Nematostella***	***Hydra***
MCPsignal	PF00015	Methyl-accepting chemotaxis protein (MCP) signaling domain	2	1	15	7
wnt	PF00110	wnt family	15	15	29	11
G-alpha	PF00503	G-protein alpha subunit	13	18	34	19
RGS	PF00615	Regulator of G protein signaling domain	10	11	13	13
G-gamma	PF00631	GGL domain	3	3	4	2
HAMP	PF00672	HAMP domain	0	0	6	0
DIX	PF00778	DIX domain	3	2	4	1
STAT_alpha	PF01017	STAT protein, all-alpha domain	1	1	2	0
CheW	PF01584	CheW-like domain	0	0	1	0
Hpt	PF01627	Hpt domain	0	0	1	0
Cbl_N	PF02262	CBL proto-oncogene N-terminal domain 1	1	1	1	1
Dishevelled	PF02377	Dishevelled specific domain	1	0	0	0
Cbl_N2	PF02761	CBL proto-oncogene N-terminus, EF hand-like domain	1	1	0	1
Cbl_N3	PF02762	CBL proto-oncogene N-terminus, SH2-like domain	1	1	0	1
STAT_bind	PF02864	STAT protein, DNA binding domain	1	1	1	1
STAT_int	PF02865	STAT protein, protein interaction domain	1	1	0	1
NPH3	PF03000	NPH3 family	0	0	0	0
Focal_AT	PF03623	Focal adhesion targeting region	1	0	1	2
Olfactory_mark	PF06554	Olfactory marker protein	0	0	0	0
Phe_ZIP	PF08916	Phenylalanine zipper	1	1	0	0
TRADD_N	PF09034	TRADD, N-terminal domain	0	0	0	0
TGF_beta	PF00019	Transforming growth factor beta like domain	7	10	9	11
FGF	PF00167	Fibroblast growth factor	14	13	14	13
PDGF	PF00341	Platelet-derived growth factor (PDGF)	2	0	0	1
TGFb_propeptide	PF00688	TGF-beta propeptide	6	11	6	10
IL2	PF00715	Interleukin 2	0	0	0	0
IL4	PF00727	Interleukin 4	0	0	0	0
PTN_MK_C	PF01091	PTN/MK heparin-binding protein family, C-terminal domain	0	0	0	0
GM_CSF	PF01109	Granulocyte-macrophage colony-stimulating factor	0	0	0	0
IL7	PF01415	Interleukin 7/9 family	0	0	0	0
IL5	PF02025	Interleukin 5	0	0	0	0
IL3	PF02059	Interleukin-3	0	0	0	0
IL12	PF03039	Interleukin-12 alpha subunit	0	0	0	0
Rabaptin	PF03528	Rabaptin	1	1	1	0
PDGF_N	PF04692	Platelet-derived growth factor, N terminal region	0	0	0	0
AMH_N	PF04709	Anti-Mullerian hormone, N terminal region	0	0	0	0
PTN_MK_N	PF05196	PTN/MK heparin-binding protein family, N-terminal domain	0	0	1	0
CSF-1	PF05337	Macrophage colony stimulating factor-1	0	0	0	0
PSK	PF06404	Phytosulfokine precursor protein (PSK)	0	0	0	0
IL11	PF07400	Interleukin 11	0	0	0	0
