# Supplementary material for: A Snapshot of a Coral “Holobiont”: A Transcriptome Assembly of the Scleractinian Coral, Porites, Captures a Wide Variety of Genes from Both the Host and Symbiotic Zooxanthellae
Source: PLoS One. 2014 Jan 15;9(1):e85182. doi: 10.1371/journal.pone.0085182 (PMC3893191; doi:10.1371/journal.pone.0085182)
Supplement: Figure S3 — Analysis of GO term enrichment of the “molecular function” category for Porites and Symbiodinium contigs. GO terms containing at least 100 sequences are shown. The Y-axis represents proportions of contigs in each category of GO-assigned contigs (Porites: 9806, Symbiodinium: 9147). Blue bars indicate Porites contigs. Red bars indicate Symbiodinium contigs. (PDF) [file pone.0085182.s003.pdf]

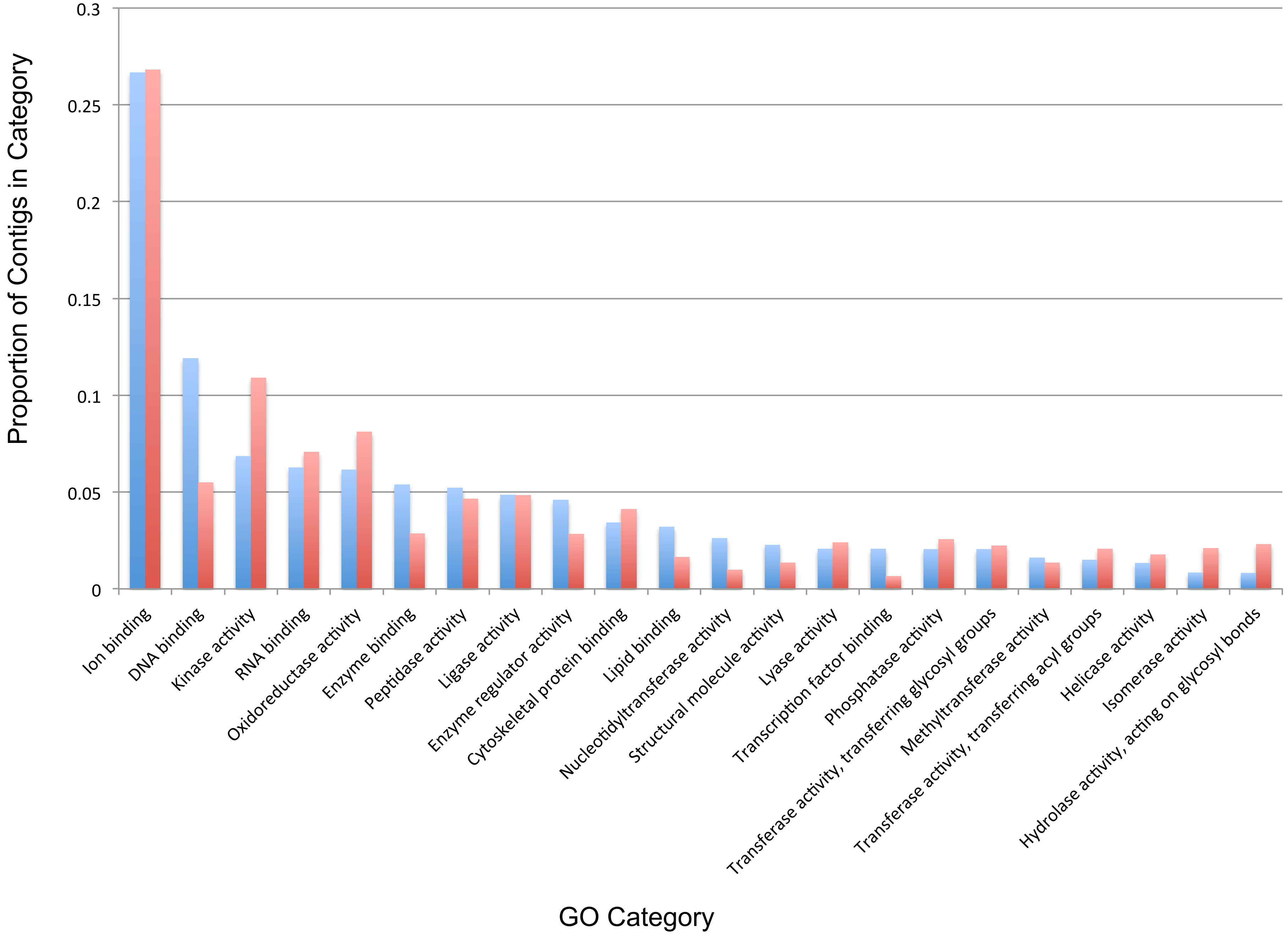
Proportion of Contigs in Category
0.3
0.25
0.2
0.15
0.1
0.05
0
Ion binding
DNA binding
Kinase activity
RNA binding
Oxidoreductase activity
Enzyme binding
Peptidase activity
Ligase activity
Enzyme regulator activity
Cytoskeletal protein binding
Lipid binding
Nucleotidyltransferase activity
Structural molecule activity
Lyase activity
Transcription factor binding
Phosphatase activity
Transferase activity, transferring glycosyl groups
Methyltransferase activity
Transferase activity, transferring acyl groups
Helicase activity
Isomerase activity
Hydrolase activity, acting on glycosyl bonds
GO Category

Figure S3
